# Cement-Augmented Versus Conventional Pedicle Screws in Surgical Management of Osteoporotic Vertebral Fractures

**DOI:** 10.7759/cureus.76091

**Published:** 2024-12-20

**Authors:** Daigo Arimura, Hiroki Wakiya, Shunsuke Katsumi, Shintaro Obata, Akira Shinohara, Mitsuru Saito

**Affiliations:** 1 Department of Orthopedic Surgery, Jikei University School of Medicine, Tokyo, JPN

**Keywords:** cement-augmented pedicle screw, cement leakage, osteoporosis, osteoporotic vertebral fractures, posterior fixation

## Abstract

Objective: Cement-augmented pedicle screws (CAPS) are expected to have fewer complications than conventional pedicle screws (PS), including less risk of postoperative screw loosening and loss of correction. However, use of CAPS has been associated with a risk of other complications, such as cement leakage. In this study, we investigated the usefulness of CAPS for osteoporotic vertebral fractures (OVF) by comparing its surgical outcomes with those of PS.

Methods: The study included 46 patients (PS group, n=29; CAPS group, n=17) who underwent posterior fixation surgery for OVF at our hospital or an affiliated hospital between August 2019 and August 2023 and were followed up for 12 months postoperatively. We collected background information on each patient, including age, sex, body mass index, diagnosis, and whether they were receiving osteoporosis treatment. We also examined the level of the lesion, the range of fixation (1-1, 2-2, 3-3), whether posterior bone grafting was performed, whether anterior column reconstruction was performed, and the corrected angle. We compared surgery-related factors, including the rate of revision surgery within 12 months postoperatively, adjacent vertebral fracture, screw loosening, and loss of angle correction at 12 months postoperatively between the two groups. We also evaluated cement leakage in the CAPS group. Logistic and linear regression analyses were used to evaluate the factors associated with each outcome.

Results: The Elixhauser Comorbidity Index value was significantly higher in the PS group, (P=0.042), but there were no significant differences between the groups in other factors. Cement leakage was confirmed in seven of the 17 cases in the CAPS group, all of which were asymptomatic. There was no significant difference between the groups in the incidence of adjacent vertebral fractures, screw loosening, or loss of correction. There was also no significant difference in the incidence of early revision surgery between the PS group (7/36 cases) and the CAPS group (7/40 cases). The risk of adjacent vertebral fracture was shown to be significantly higher at the thoracic level than at the thoracolumbar level (odds ratio 34, P=0.043). The risk of thoracic vertebral fracture was significantly higher than that of thoracolumbar vertebral fracture in cases with loss of angle correction (B=1.312, P=0.029). No significant risk factors for screw loosening were identified.

Conclusions: There was no significant difference in surgical outcomes between CAPS and conventional PS when used in posterior fusion surgery for thoracolumbar fractures. However, use of CAPS was associated with a risk of cement leakage. Therefore, the indications for its use should be carefully considered. Long-term, large-scale prospective studies are needed to assess the usefulness of CAPS in more detail.

## Introduction

Osteoporosis is a major public health problem worldwide that is increasing with population aging [1]. It is a systemic skeletal disease that causes a decrease in bone mineral density (BMD), deterioration of bone microstructure and reduction in bone strength and is associated with an increased risk of low-energy and fragility fractures [2]. Osteoporotic vertebral fractures (OVF) are common in the elderly and are estimated to affect approximately 50 million people worldwide [3]. These fractures have an adverse impact on the mobility of the spine, causing severe pain and functional disability and significantly reducing patients' quality of life [4]. Stabilization of the fractured vertebral body and pain relief are essential in the treatment of OVF. Posterior fixation with conventional pedicle screws (PS) is the usual method used to treat OVF [5]. However, in patients with osteoporosis, where bone quality is significantly reduced, the screws may not be adequately fixed, and complications such as screw loosening [6] and kyphosis of the proximal junction [7] may occur.

As a solution to these problems, cement-reinforced pedicle screws with a window [8], hereafter referred to as cement-augmented pedicle screws (CAPS), are gradually entering clinical practice [9]. The advantage of this screw is that the contact surface between the bone and the screw is increased by injecting polymethylmethacrylate (PMMA) cement, thereby improving fixation strength [10]. Cement-impregnated screws have high pull-out strength [11], which reduces the risk of screw loosening and improves fixation [12]. However, cement augmentation of pedicle screws may be associated with complications, such as leakage and embolism [13], which should not be ignored.

The aim of this study was to compare the surgical outcomes of posterior fusion using PS with those of CAPS in patients with OVF and to evaluate the hypothesis that there are advantages that outweigh the risks of CAPS.

## Materials and methods

Patients who underwent posterior fusion surgery for OVF using PS or CAPS at Jikei University Hospital or an affiliated hospital between August 2019 and August 2023 were included in the study. The study protocol was approved by the Jikei University School of Medicine Ethics Committee (30-115 {9136}) and performed in accordance with the tenets of the Declaration of Helsinki. Written informed consent was obtained from all patients.

The patients underwent surgery for back pain caused by OVF that was refractory to conservative treatment or for neurological symptoms resulting from nerve compression. Patients with degenerative diseases or fractures attributable to high-energy trauma were excluded, as were those with insufficient data owing to revision surgery (within 12 months), death, or loss to follow-up.

The decision regarding the extent of fusion and the surgical technique was made at the discretion of each surgeon, but was generally in accordance with the following strategy. In addition to posterior fusion, anterior column reconstruction with vertebroplasty is always the first choice. In cases where vertebroplasty is difficult or there is vacuum degeneration between the vertebrae, vertebral replacement or anterior intervertebral fusion is performed. In cases where there are many complications due to old age or where the operation time needs to be shortened, the surgeon decides whether to perform posterior fixation surgery. The decision to perform a bone graft is also made by the surgeon. Regarding the choice of screw type, CAPS (DePuy Synthes fenestrated screw system, Raynham, MA, USA) has been available in Japan since October 2020. In our hospital, it became available in March 2022; since then CAPS is generally chosen for fractures. Even after CAPS became available, conventional PS has been used at the surgeon's discretion in some patients, such as those at high risk of cement leakage. All surgical procedures are performed by one or two specialists.

Information was collected on patient background characteristics, including age at surgery, sex, body mass index, presence of osteoporosis treatment, and comorbidities. Comorbidities were assessed using the Elixhauser Comorbidity Index [14]. Surgery-related parameters included lesion level (thoracic, thoracolumbar [T12-L1] or lumbar), fixation range (1-1, 2-2 or 3-3), posterior bone grafting, anterior reconstruction (including vertebroplasty, bone grafting, anterior vertebral fixation, and vertebral replacement), and angle correction. The primary endpoints were the incidence of adjacent fractures, screw loosening, and loss of angle correction at 12 months postoperatively. The secondary endpoints were the incidence of removal or additional surgery during the 12 months postoperatively and the presence or absence of cement leakage in the CAPS.

Patients were divided into those who underwent surgery with conventional PS (the PS group) and those who underwent surgery with CAPS (the CAPS group). Background characteristics and surgery-related parameters were compared between the two groups. The relationship between preoperative factors (including use of CAPS) and adjacent fracture, screw loosening, and loss of angle correction was evaluated.

Continuous data are reported as the mean ± standard deviation if normally distributed and as the median (interquartile range) if not normally distributed. Categorical data are expressed as the number and percentage. The unpaired t-test was used to compare normally distributed continuous data between groups and the Mann-Whitney U test was used for comparisons of nonparametric data. Fisher’s exact test was used to compare ratios. The influence of independent variables on screw loosening and adjacent fractures by 12 months postoperatively was evaluated by logistic regression analysis. Linear regression analysis was used to test whether independent variables were associated with loss of angle correction after 12 months; the loss after 12 months was ln-transformed to fit this model. All statistical analyses were performed using SPSS version 29.0.2.0 for Windows (IBM Corp., Armonk, NY, USA). A P-value of ≤0.05 was considered statistically significant.

## Results

Demographics

A total of 76 patients (PS group, n=40; CAPS group, 36 cases) underwent posterior fusion during the study period. Of these, 14 cases with insufficient data (three in the PS group and 11 in the CAPS groups), two who died (one in each study group), and 14 who underwent revision surgery within 12 months postoperatively (seven in each study group) were excluded. Finally, 12 months of postoperative data for 46 patients (PS group, n=29; CAPS group, n=17) were available for evaluation.

Comparison between groups

The patient characteristics are shown in Table 1.

**Table 1:** Comparison of patient and surgery-related factors between the PS group and the CAPS group Data were expressed as the number and percentage, mean ± standard deviation, or median [interquartile range] as appropriate. ^a^Fisher's exact test; ^b^unpaired t-test; ^c^Mann–Whitney U test. CAPS, cement-augmented pedicle screws; ECI, Elixhauser Comorbidity Index; PS, conventional pedicle screws

		PS (n=29)	CAPS (n=17)	P-value
Age		74.7±8.3	78.7±8.9	0.133^ b^
Sex				0.235^ a^
	Male	14, 48.3	5, 29.4	
	Female	15, 51.7	12, 70.6	
Body mass index		23.6±4.4	21.8±5.0	0.222^b^
Osteoporosis treatment		11, 37.9	8, 47.1	0.757^a^
ECI		0.82 [0.78, 0.82]	0.79 [0.74, 0.82]	0.042^c^
Lesion level				0.063^a^
	Thoracic	1, 3.4	1, 5.9	
	Thoracolumbar	25, 86.2	10, 58.8	
	Lumbar	3, 10.3	6, 35.3	
Fixed range				0.090^a^
	1-1	8, 27.6	9, 52.9	
	2-2	12, 41.4	7, 41.2	
	3-3	9, 31.0	1, 5.9	
Posterior bone graft (yes)		4, 13.8	4, 23.5	0.443^a^
Anterior reconstruction (yes)		23, 79.3	15, 88.2	0.691^a^
Correction angle		6.0 [2.5, 7.9]	6.0 [2.5, 9.2]	0.715^c^

The Elixhauser Comorbidity Index value was significantly higher in the CAPS group. The primary endpoints at 12 months are shown for each group in Table 2.

**Table 2:** Comparison of outcomes at 12 months postoperatively between the PS group and the CAPS group The data are expressed as the number and percentage or as the median [interquartile range] as appropriate. ^a^Fisher's exact test; ^b^Mann–Whitney U test. CAPS, cement-augmented pedicle screws; PS, conventional pedicle screws

	PS (n=29)	CAPS (n=17)	P-value
Adjacent fractures	2, 6.9	1, 5.9	>0.999^a^
Screw loosening	12, 41.4	3, 17.6	0.117^a^
Correction loss angle	6.0 [3.3, 8.5]	6.2 [2.0, 9.5]	0.698^b^
Cement leakage		7.0, 41.2	-^a^

**Figure 1:**
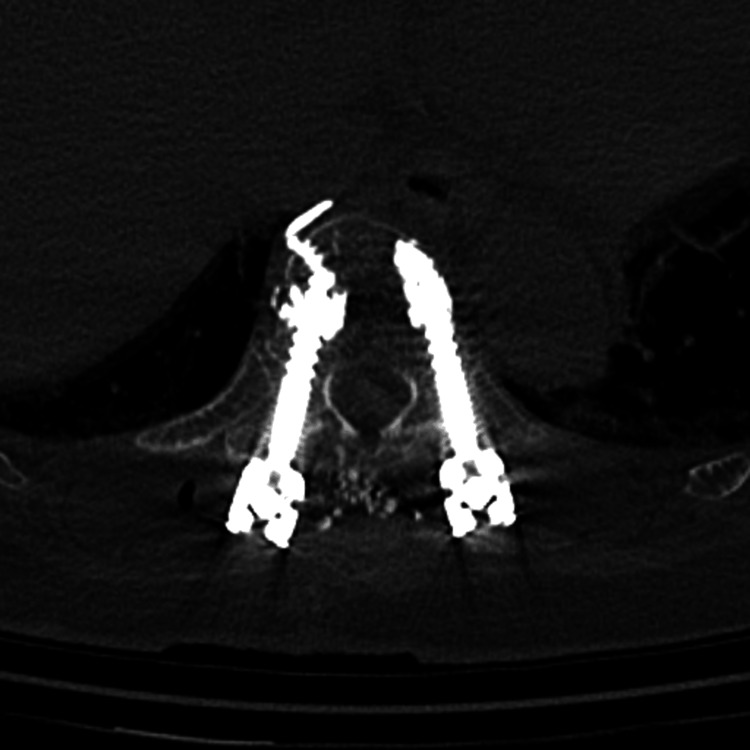
A representative image of cement leakage This cement leakage were classified as S type (leakage via segmental veins).

There was no significant difference in the incidence of adjacent fracture, screw loosening, or loss of correction angle between the PS and CAPS groups at 12 months postoperatively. In all cases, we perform CT scans on the day of surgery or the following day to check the position of the implant and determine whether there is cement leakage. In the CAPS group, we observed cement leakage in seven cases. All cases of cement leakage were classified as S type (leakage via segmental veins) according to a classification method used previously in the literature [13]. A representative example is shown in Figure 1.

All patients with cement leakage had no symptoms and were managed with follow-up observation only. There was no significant difference in the rate of revision surgery between the groups (Table 3).

**Table 3:** Comparison of the rate of revision surgery between the PS group and the CAPS group The data are expressed as the number and percentage. ^a^Fisher's exact test. CAPS, cement-augmented pedicle screws; PS, conventional pedicle screws

	PS (n=40)	CAPS (n=36)	P-value
Revision surgery	7, 17.5	7, 19.4	>0.999^a^

Logistic regression analysis for loosening 12 months postoperatively

Logistic regression analysis did not identify any variables that had a significant impact on the risk of screw loosening at 12 months postoperatively (Table 4).

**Table 4:** Logistic regression analysis for loosening by 12 months postoperatively CAPS, cement-augmented pedicle screws; CI, confidence interval; ECI, Elixhauser Comorbidity Index; n.c., not calculated; OR, odds ratio; PS, conventional pedicle screws

		Univariate analysis		
		OR	95% CI	P-value
Age (per 1 year)		1.060	0.975, 1.152	0.172
Sex, female (vs. male)		0.722	0208, 2.508	0.608
Body mass index (per 1)		0.999	0.874, 1.143	0.994
Osteoporosis treatment, yes (vs. no)		0.607	0.168, 2.196	0.447
ECI (per 0.1)		1.303	0.318, 5.345	0.713
Lesion level				
	Thoracic	n.c.		
	Thoracolumbar	1.000	ref	
	Lumbar	0.958	0.203, 4.523	0.957
Fixed range				
	1-1	1.000	ref	
	2-2	3.394	0.725, 15.897	0.121
	3-3	3.111	0.527, 18.382	0.210
Posterior bone graft, yes (vs. no)		0.245	0.027, 2.203	0.209
Anterior reconstruction, yes (vs. no)		1.560	0.275, 8.843	0.615
Correction angle (per 1)		0.963	0.844, 1.100	0.580
CAPS (vs. PS)		0.304	0.071, 1.293	0.107

Logistic regression analysis for adjacent fractures 12 months postoperatively

The results of logistic regression analysis for adjacent fractures at 12 months postoperatively are shown in Table 5. There was a significant difference in the risk of thoracic vertebral fracture compared with that of thoracolumbar vertebral fracture (odds ratio 34.000, 95% confidence interval 1.122-1030.416, P=0.043). There was no significant difference in any of the other variables.

**Table 5:** Logistic regression analysis for adjacent fractures 12 months postoperatively CAPS, cement-augmented pedicle screws; CI, confidence interval; ECI, Elixhauser Comorbidity Index; n.c., not calculated; OR, odds ratio; PS, conventional pedicle screws; ref, reference

		Univariate analysis		
		OR	95% CI	P-value
Age (per 1 year)		1.035	0.889, 1.205	0.655
Sex, female (vs. male)		1.440	0.121, 17.122	0.773
Body mass index (per 1)		0.852	0.610, 1.189	0.345
Osteoporosis treatment, yes (vs. no)		n.c.		
ECI (per 0.1)		17.178	0.077, 3854.312	0.303
Lesion level				
	Thoracic	34.000	1.122, 1030.416	0.043
	Thoracolumbar	1.000	ref	
	Lumbar	4.250	0.239, 75.470	0.324
Fixed range				
	1-1	1.000	ref	
	2-2	0.417	0.034, 5.057	0.492
	3-3	n.c.		
Posterior bone graft, yes (vs. no)		n.c.		
Anterior reconstruction, yes (vs. no)		n.c.		
Correction angle (per 1)		0.823	0.566, 1.196	0.307
CAPS (vs. PS)		0.844	0.071, 10.064	0.893

Linear regression analysis for ln-transformed corrected loss 12 months postoperatively

The results of the linear regression analysis for ln-transformed loss of angle correction by 12 months postoperatively are shown in Table 6. There was a significant difference in the risk of thoracic vertebral fracture compared with that of thoracolumbar vertebral fracture (B=1.312, β=0.326, P=0.029). There was no significant difference in any of the other variables.

**Table 6:** Linear regression analysis for ln-transformed corrected loss at 12 months postoperatively B, regression coefficients; β, standardized regression coefficients; CAPS, cement-augmented pedicle screws; ECI, Elixhauser Comorbidity Index; PS, conventional pedicle screws; SE, standard error

		Univariate analysis				
		B	SE	β	t	P-value
Age (per 1 year)		0.015	0.014	0.158	1.062	0.294
Sex, female (vs. male)		-0.195	0.250	-0.117	-0.780	0.440
Body mass index (per 1)		-0.003	0.027	-0.016	-0.107	0.915
Osteoporosis treatment, yes (vs. no)		-0.218	0.249	-0.131	-0.874	0.387
ECI (per 0.1)		0.104	2.750	0.006	0.038	0.970
Lesion revel						
	Thoracic	1.312	0.582	0.326	2.253	0.029
	Thoracolumbar	ref				
	Lumbar	-0.091	0.299	-0.044	-0.303	0.763
Fixed range						
	1-1	ref				
	2-2	-0.036	0.283	-0.021	-0.126	0.900
	3-3	0.055	0.338	0.027	0.162	0.872
Posterior bone graft, yes (vs. no)		0.221	0.325	0.102	0.679	0.501
Anterior reconstruction, yes (vs. no)		0.043	0.327	0.020	0.131	0.897
Correction angle (per 1)		0.002	0.025	0.015	0.097	0.923
CAPS (vs. PS)		-0.224	0.254	-0.132	-0.881	0.383

## Discussion

CAPS are an effective fixation strategy that is intended to improve pedicle screw stability in patients with osteoporosis and may increase fixation strength [15,16]. CAPS are also expected to stabilize the spine immediately and help to lessen postoperative pain [17]. However, they must be used carefully because of the risk of cement leakage. In this study, we compared the surgical outcomes of CAPS with those of conventional PS in patients with osteoporotic vertebral fracture and assessed whether CAPS has benefits that outweigh the risks.

At 12 months postoperatively, there were two cases of adjacent vertebral fracture in the PS group and one case in the CAPS group, with no significant difference between the groups. The loss of angle correction was similar in the PS group and the CAPS group (6.0 degrees vs 6.2 degrees).

Correction angles were also similar in both groups (6.0 degrees vs. 6.0 degrees). In OVF surgery, correction was basically performed in situ in the prone position and no active correction was performed. Therefore, the superiority of CAPS was not observed.

Although there was no statistically significant difference in the number of loose screws, there was a trend for fewer loose screws in the CAPS group than in the PS group (3 vs 12). There was also no significant difference in the incidence of early revision surgery (7/40 cases in the CAPS group and 7/36 cases in the PS group). The risk of adjacent fractures was significantly higher at the thoracic (T1-T11) level than at the thoracolumbar (T12-L1) level (odds ratio 34, P=0.043). Furthermore, the risk of thoracic fractures was significantly higher in relation to the loss of angle correction (B=1.312, P=0.029). However, no significant risk factors for screw loosening were identified, including screw type.

In a study by Wang et al., which included patients with degenerative diseases, pain reduction and functional recovery at six months postoperatively (indicated by Oswestry Disability Index and Japanese Orthopedic Association scores) were significantly better in the CAPS group than in the PS group; moreover, the incidence of screw loosening was significantly lower (1.04%) in the CAPS group, indicating improved fixation strength [12]. A meta-analysis by Yagi et al. also found that use of CAPS significantly reduced the frequency of screw loosening and the need for revision surgery and improved postoperative spinal stability [18]. These findings suggest that CAPS may be an effective option for the treatment of OVF. However, in the present study, although there was a trend towards a lower incidence of screw loosening in the CAPS group, the between-group difference was not statistically significant. There was also no significant between-group difference in loss of angle correction, the adjacent vertebral fracture rate, or the frequency of revision surgery. One possible reason for the lack of clear evidence of the superiority of CAPS is that our study only targeted OVF and was performed in a population with a particularly high proportion of elderly patients (74.7 ± 8.3 years in the PS group and 78.7 ± 8.9 years in the CAPS group). In vitro biomechanical experiments by Zhuang et al. found that when BMD was less than 0.6 g/cm², there was a high risk of loosening even when the screws were reinforced with PMMA [19]. Although BMD was not measured in detail in our study population, it is possible that the effect of CAPS on fixation was not fully demonstrated because of bone fragility. However, the meta-analysis by Yagi et al., which included patients aged 18 years or older with degenerative or traumatic diseases, found that the effectiveness of CAPS was more pronounced in patients who were relatively young with maintained BMD [18]. In addition, the two cases diagnosed with thoracic vertebral fractures in our study had fractures at T10 and T11 with a fusion range of 1-1. In these cases, the fracture occurred at a site adjacent to the caudal vertebra of the fixed end, which may have caused the loss of correction. However, there were only two thoracic vertebrae fracture cases and both were close to the thoracolumbar transition zone, so it is difficult to conclude that thoracic vertebrae fractures are at higher risk of adjacent fractures and loss of correction than thoracolumbar vertebrae fractures. Larger data sets and longer-term follow-up are required in future studies.

Care must be taken when cementing PS because of the risk of complications, such as cement leakage and associated embolization [9,13,16,20]. Sites of cement leakage have been reported within the spine (epidural and intradural), epidural veins, inferior vena cava, and pulmonary artery (leakage rate 3.5-23%), which can cause nerve damage [21] as well as right heart failure and respiratory failure if an embolism occurs in the right atrium, pulmonary artery, lung, or heart [22,23]. Therefore, it is necessary to be fully aware of the risks associated with injection of cement itself. In this study, the incidence of cement leakage was 33% (12 of 36 cases), and all cases were asymptomatic. In the literature, the incidence of cement leakage varies widely from 10% to 80% depending on the study [13]. In the meta-analysis by Yagi et al., the combined data from 16 studies reporting cement leakage showed a leakage rate of 34.36% [18], which is similar to the rate in our present study. However, not all cases of leakage cause clinical symptoms, and in patients treated with an augmented PS, the rate of symptomatic cement leakage was reported to be 5.5%, that of asymptomatic leakage was 66.7%, and that of anaphylactic reactions was 1.2% [20]. Furthermore, some studies have suggested that cement augmentation may affect adjacent vertebral loading and increase the risk of new adjacent vertebral fractures, particularly in patients with osteoporosis [24]. A finite element analysis study by Kim et al. found that when the volume of PMMA filling exceeded 30% of the vertebral body volume, the hardness of normal bone was exceeded, resulting in an increased risk of adjacent vertebral fracture [25].

Several measures have been reported to reduce the risk of cement leakage when using CAPS. First, it is thought that adjusting the screw insertion angle more inward may reduce this risk [13]. In addition, patients with very low BMD tend to have a higher risk of cement leakage [26], and more careful manipulation is required when injecting cement in these cases. The amount of cement administered is a risk factor for cement leakage [13], and it has been reported that reducing the amount of cement used from 3 ml to 1 ml has little effect on fixation strength [9]. Therefore, the amount of bone cement used for each screw was set at 1 ml or less to reduce the risk of leakage during cement injection. It is also believed that partially maintaining the patient's positive end-expiratory pressure at 15 cmH₂O during injection of cement may help to reduce the risk of cement leakage [27]. It is thought that nerve monitoring in combination with control of positive end-expiratory pressure can help to detect and avoid nerve damage caused by cement leakage at an early stage [28]. Although cement leakage is relatively common, cases resulting in serious complications are rare. By understanding the risks associated with injection of cement and using appropriate techniques and management, it is possible to minimize the risk of this complication and maximize the effectiveness of the procedure.

This study has several limitations. First, the sample size was relatively small, and the numbers of patients in the PS and CAPS groups were not well balanced, which may have affected the statistical power of the study. It is also important to remember that the level of activity after surgery will be different for each patient. In addition, the short follow-up period of only one year should also be taken into account when interpreting the results of this study. Future research should use larger, more balanced samples with longer follow-up periods. Second, considering the retrospective nature of the study, the possibility that bias in patient selection or incomplete data collection may have affected the results cannot be ruled out. A prospective randomized controlled trial is needed to assess causality in more depth.

Third, the relatively short follow-up period did not allow for full evaluation of long-term maintenance of screw fixation or complications related to injection of cement. Longer-term follow-up data are essential to evaluate the long-term efficacy and safety of CAPS. Fourth, it is possible that differences in surgeon skill and experience may have influenced their cement injection technique. In the future, it will be necessary to establish a standardized protocol for the amount and method of cement injection, which will improve the reproducibility of treatment and its outcomes. Finally, there was no detailed analysis of the impact of the degree of progression of osteoporosis and the patient's general condition on surgical outcomes. It is important to evaluate BMD and general health in more detail and to clarify how these factors affect the success of screw fixation and postoperative outcomes. Further research is needed to select the optimal treatment method for each patient.

## Conclusions

In this study, we compared the surgical outcomes of posterior fusion using CAPS and conventional PS for osteoporotic vertebral fractures in the elderly. Although there was a trend toward less screw loosening with CAPS, there was no significant difference between the two groups in the incidence of adjacent vertebral fractures or loss of correction. Use of CAPS is associated with cement leakage, and there is concern about the effect on surrounding tissue and blood vessels, so care is required when using these screws. Our findings suggest that when performing vertebral fracture surgery in elderly patients, if the angle of screw insertion is more lateral than intended, the risk of cement leakage should be considered and cement injection avoided. In the future, it will be important to accumulate further evidence on the long-term efficacy and safety of CAPS in larger, long-term prospective studies and to select appropriate indications and make technical improvements. It will also be necessary to find ways to minimize the risk of cement leakage. It is hoped that the selection of screws and optimization of treatment methods, taking into account the characteristics of individual patients, will contribute to improved outcomes in the treatment of osteoporotic vertebral fractures.
